# Could Contracts between Pharmaceutical Firms and French Veterinarians Bias Prescription Behaviour: A Principal-Agency Theory Approach in the Context of Oligopolies

**DOI:** 10.3390/antibiotics10020176

**Published:** 2021-02-10

**Authors:** Didier Raboisson, Ahmed Ferchiou, Tifenn Corre, Sylvain Perez, Pierre Sans, Guillaume Lhermie, Marie Dervillé

**Affiliations:** 1 CIRAD, UMR ASTRE, Montpellier, France, ASTRE, CIRAD, INRAE, Univ Montpellier, Montpellier, Université de Toulouse, ENVT, 31300 Toulouse, France; ahmed.ferchiou@envt.fr (A.F.); sylvain.perez_13@envt.fr (S.P.); guillaume.lhermie@envt.fr (G.L.); 2 US ODR, INRAE, 31320 Auzeville-Tolosane, France; tifenn.corre@inrae.fr; 3 UR 1303 ALISS, INRAE, 94205 Ivry-sur-Seine, France; pierre.sans@envt.fr; 4 Université de Toulouse, LEREPS, ENSFEA, 31300 IEP de Toulouse, France; marie.derville@ensfea.fr

**Keywords:** drugs, veterinarian, pharmaceutical firm, contract

## Abstract

In France, veterinarians can both prescribe and deliver veterinary medicines, which is a questionable situation from the perspective of antimicrobial use (AMU) reduction to avoid antimicrobial resistance (AMR). This situation places veterinarians in direct commercial relationships with the pharmaceutical industry as purchase contracts are signed between veterinarians and pharmaceutical companies. The aim of the present work is to analyse the relationships between veterinarians and pharmaceutical firms in the oligopoly market context of French veterinary medicine to determine whether the prescription behaviour of practitioners can be biased by joint prescription and delivery. Therefore, we develop an analysis based on principal-agent theory. Contracts between pharmaceutical companies and veterinarians during the 2008–2014 period were analysed based on 382 contracts related to 47 drugs belonging to eight main pharmaceutical firms (2320 observations). The price per unit after rebate of each drug and contract was calculated. The descriptive analysis demonstrated high disparity among the contracts across pharmaceutical firms with regard to the provisions of the contracts and how they are presented. Then, linear regression was used to explain the price per unit after rebate based on the explanatory variables, which included the yearly purchase objective, year, type of drug and type of rebate. The decrease in price per unit after rebate for each extra €1000 purchase objective per drug category was established to be €0.061 per 100 kg body weight for anticoccidiosis treatments, €0.029 per 100 kg body weight for anti-inflammatories, €0.0125 per 100 kg body weight and €0.0845 per animal for antiparasitics, and €0.031 per animal for intramammary antimicrobials. Applying agency theory reveals that veterinarians can be considered agents in the case of monopolistic situations involving pharmaceutical firms; otherwise, veterinarians are considered principals (oligopolistic situations in which at least several medicines have similar indications). The present study does not provide evidence suggesting that joint prescription and delivery may introduce any potential prescription bias linked to conflicts of interest under the market conditions during the 2008–2014 period.

## 1. Introduction

Antimicrobial resistance (AMR) observed in humans is accentuated by the use of AMs in humans and animals. AMR in animals is mostly the result of the use of AMs in animals [1]. Over several decades, inappropriate medical prescription and administration have been noted as primary factors contributing to this global issue [1]. Thus, many strategies, such as the ban of the use of antimicrobial growth promotors and the establishment of surveillance systems, have been adopted to cope with this issue [2]. France also participates in this movement through its Ecoantibio plan, which reduced the total consumption of antimicrobials in livestock by up to 37% from 2012 to 2017 [3]. This decrease represented a 75% decrease in fluoroquinolones and an 81% decrease in the last generation of cephalosporins [4].

**Figure 1:**
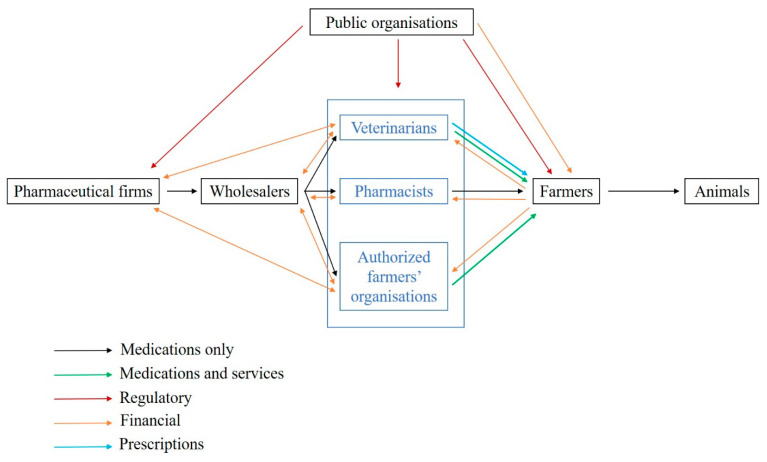
Prescription and delivery system of veterinary medicines in France (adapted from [13]). Legend: This figure represents the supply chain of veterinary medicines (in black), including delivery to farmers by veterinarians, pharmacists and authorised farmer organisations. The prescription flow between veterinarians and farmers is indicated in blue. The star represents the contractual and commercial relationships between veterinarians and pharmaceutical firms that are studied in the present work.

In France, veterinary medicines can only be prescribed by veterinarians, and medicine delivery is restricted to veterinarians, pharmacists and farmer organizations depending on the medicine class (Figure 1). The medicine supply chain comprises (i) pharmaceutical firms, which can subcontract medicine production, (ii) wholesalers, (iii) veterinarians and other actors allowed to deliver medicines and iv) farmers or animal owners [5]. More than 80% of medicine delivery is performed by veterinarians despite some variations between livestock systems and species. A recent study highlights that the share of income raised from medicine delivery in France is on average 30–40% for companion animals and 60–80% for large animals [6]. There is increasing concern regarding conflicts of interest due to the simultaneous prescription and delivery of medicines by veterinarians [7,8]. However, in European countries where prescription and delivery are decoupled, no systematic changes in the pattern of AM use (AMU) have been observed [9] likely because multiple factors influence the end-user of veterinary medicine [5], which is a regulated product in most countries. The price of AMs is known to be a key driver of use in veterinary medicine [10,11]. In human medicine, a link among AM price, AMU and increased AMR has been demonstrated. For instance, in Denmark, the increase in the number of medicines containing ciprofloxacin (from 3 to 10) was associated with a decrease in medicine prices by 53%. The proportion of urinary *E. coli* resistant to ciprofloxacin increased by 200% in the following 4 years [12].

The induced demand for medicines by patients and farmers is not well understood in both human and veterinary medicine. Demand is influenced by disease risk management and prevention practices, but pharmaceutical firms may attempt to influence prescription behaviour through ads, communications and even gifts. In the case of coupled prescription and delivery, prescribers may also be influenced during prescription by the delivery facility or interest. A link between a prescriber’s tendency to prescribe medicines that are more profitable to him/her and medicine delivery rebates has been shown in China [14]. On the one hand, the relationship between prescribers and pharmaceutical firms reportedly encourages inappropriate usage to increase medical costs and favour the propagation of resistance [15] through asymmetric information [16]. Prescribers who frequently meet with a pharmaceutical salesperson tend to (i) more easily prescribe a newly arrived medicine and (ii) overuse/overprescribe medicines due to the ease of his/her permission for a patient’s request for a prescription, even if the prescription is not medically advisable [17,18]. The pharmaceutical industry is also known to use the push strategy (e.g., promotions, funding, and sponsorship) in its relationship with prescribers [15,19], even though these actions tend to be regulated in many countries. On the other hand, the close relationships between the pharmaceutical industry and prescribers (i) help prescribers access information in some areas, even if bias is present [20,21], (ii) improve innovation due to the positive impact of sharing information [22], and iii) optimize supply chain management [23]. Relationship marketing remains a primary driver of sales in the pharmaceutical industry [24].

A key question is to determine whether prescription behaviour is influenced by joint prescription and delivery and/or the contractual relationship between veterinarians and pharmaceutical firms. Prescription freedom is a key issue in public health. A recent study observed a change in prescription behaviour in the case of new medicines available on the market, but medicine prescription substitution was observed only within the same medicine category of the AM family [5], suggesting that in this situation, joint prescription and delivery had no impact. Therefore, prescribers represent a strong filter to access to veterinary medicines.

The principal-agent approach is appropriate for better understanding the relationship between veterinarians and the pharmaceutical industry and evaluating whether their commercial relationships may bias prescription behaviour. We hypothesize that the veterinarian retains bargaining power (i.e., the veterinarian is the principal, and the pharmaceutical company is the agent), even in a situation of oligopoly (few companies selling medicines on the market) for a given medicine. The commercial relationship between veterinarians and pharmaceutical firms is based on annual contracts defining at least the quantity and prices of medicines and the rebate system developed by the pharmaceutical companies. The central argument of contract theory [25] is that a given good will not be exchanged at the same price if agents encounter transaction costs, if they can enjoy informational advantages or if non redeployable investments must be made (i.e., specific assets). The rules of a Walrasian market will then not be followed. To render their activities compatible and share the value surplus created, agents sign contracts that limit their behaviour and establish coordination mechanisms based on mutual obligations [26]. Veterinarians mainly sign contracts to decrease the purchase price of medicines (through rebates), while pharmaceutical firms mainly sign contracts to plan their annual sales. Another indirect benefit to veterinarians of signing contracts is a reduction in information asymmetry regarding medicine prices as they can compare prices and offers. For pharmaceutical firms, contracts also prevent changes in the choice of medicines mostly delivered by veterinarian during the year.

The aim of the present work is to define the principal and agent situations of veterinarians and pharmaceutical firms in the oligopoly market context of veterinary medicines and determine whether the prescription freedom of practitioners can be influenced by the situation of joint prescription and delivery. Therefore, French pharmaceutical contracts between pharmaceutical companies and veterinarians during the 2008–2014 period were analysed. Then, an agent-principal approach perspective was applied.

## 2. Materials and Methods

### 2.1. Data

**Figure 2:**
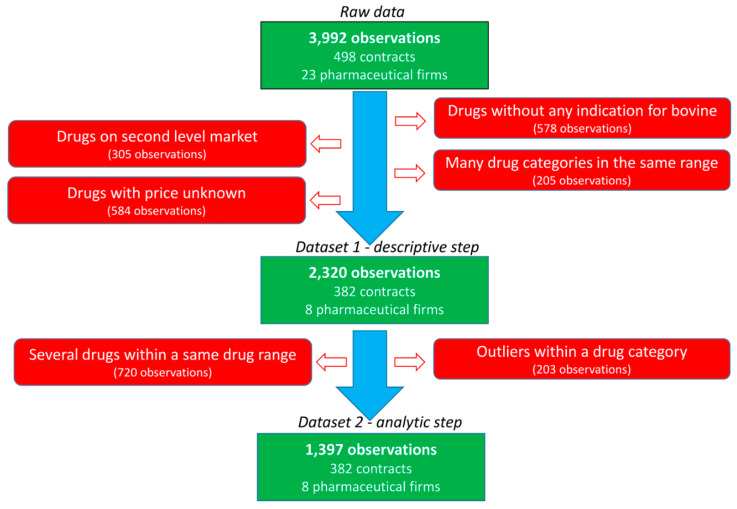
Flowchart of the data selection.

Thirty French veterinarian offices were randomly contacted to obtain their purchasing contracts with pharmaceutical firms during the 2008–2014 period. The data of five veterinary independent offices and three veterinary groups pooling their purchases were collected. All practices include both companion and farm production animal activities with various shared activities. Large variation is observed in the size of the office (one to several veterinarians per office) and the yearly revenue of the office. For inclusion, the purchase contracts should specify the medicine or group of medicines to be purchased, the objective of the purchases required for the veterinarian to obtain the rebate and the rebate provided by the pharmaceutical firm (in absolute value or percentage) if the purchase objective is achieved. In total, 498 contracts, 23 pharmaceutical firms and 125 medicines were included in the raw data (Figure 2). Most contracts were related to bovine production and restriction on this species leaded to 382 contracts related to 8 main pharmaceutical companies. The following categories of veterinary medicine were created, and data not belonging to these categories were excluded: AMs, antiparasitics (APs, i.e., pest control), anti-inflammatories (AIs) and vaccines (VACs). The final dataset included 382 contracts, 47 veterinary medicines and 2320 observations. Each medicine was coded according to the company (C1 to C8) and the medicine (P1 to P47), resulting in a combination from C1P1 to C8P47. For each medicine, the price of the medicine when the veterinarian bought the medicine from the wholesaler (i.e., before the rebate from the pharmaceutical firm) was selected.

Then, a database including the following variables was created: veterinary office, firm, year (of the contract), range (of the medicine, i.e., how the medicines were grouped in the contract), medicine name, yearly revenue from the veterinarian office of each firm, duration of the contract (quarterly, biannually, or annually), monetary purchase objective to obtain the rebate, type of rebate (per medicine, per range or global as defined below), rebate value in percentage, price of the medicine, type of medicine (parenteral administration following body weight dosage (PerBW), intramammary syringe (SYR), VAC or per animal fixed dose (DOSE)), and category of medicine (AMs, APs, AIs, or VACs). The types and categories of medicines are defined as follows: VAC are DOSE, AIs are PerBW, AMs are PerBW or SYR, and APs are PerBW or DOSE. When the rebate was indicated in whole value or free units, it was converted into a percentage of the rebate for a given objective. Three types of rebates were defined. When a medicine was explicitly mentioned in a contract (with a purchase objective and a rebate), the type of rebate was defined as the medicine. When a group of medicines was mentioned in a contract (with a purchase objective and a rebate), the type of rebate was defined as the range. In a given year, the first rebate applied to only one medicine when the purchase objective linked to this medicine is met, and the second rebate applied when the purchase objective defined for a range of medicines is met. When the rebate was given when the objectives of both the medicine and range of medicines were achieved, the rebate type was defined as global. To allow for a comparison of the contracts, a standardization of the duration was applied since 67% of the contracts were based on full years. For the same medicine, many presentations were available on the market, and the price per ml differed. Because the contract did not specify the presentation of the medicine, the combination of the presentations expected to be purchased to achieve the purchase objective was defined as the same share indicated in central average selling. When the purchase objective to be achieved to reach the rebate was defined for multiple medicines, the share of medicines was defined as equal, except for if the share was defined in the contract. When the purchase objective was defined for multiple medicines belonging to various types of medicines (such as parenteral administration following weight dosage and vaccines), these medicines were excluded since their prices cannot be standardized as explained below.

### 2.2. Price per Unit after Rebate (PUR)

To standardize how contracts may influence the final medicine price paid by a veterinarian, the price per unit of the medicine after rebate (PUR) was calculated for a treatment of 100 kg BW of an animal (parenteral administration medicines) or treatment per animal (per animal fixed dose, vaccines, intramammary syringes, etc.).

The weight of the animal treated (WAT, kg) with a given medicine was calculated using Equation (1) as follows:

WAT = Qty/Dose(1)

where Qty is the quantity of the active substance per packaging unit (mg/g or mg/mL), and dose is the dose regimen to be administered (mg or IU per kg BW); the dose was obtained from the Summary of Product Characteristics (SPC; https://www.ema.europa.eu/en). For ambiguous situations, the guidelines of the French National Veterinary Medicine Agency (ANSES) were followed. When the treatment duration was an interval, the longest duration was selected [4]. For instance, when the dose varied across species, the bovine dose was maintained.

Then, the yearly quantity of BW to be treated with the yearly contract (WAT_Contract) was calculated using Equation (2) as follows:

WAT_contract (kg) = WAT × Objective/Price(2)

where Objective is the purchase objective (€) mentioned in the contract, and price (€) is the price of the medicine.

Then, PUR was expressed in euros per 100 kg BW treated using Equation (3) as follows:

PUR (€/100 kg BW) = (Objective − Rebate)/WAT_contract × 100(3)

where Rebate is the absolute rebate (objective multiplied by the rebate value in percentage).

For intramammary syringes, PUR was calculated for the whole treatment of mastitis as indicated in the SPC. For dry-off, one treatment per teat was considered as follows:

Nb_Trt = Nb_Syr _Pack/Nb_Syr_Trt(4)

where Nb_Trt is the number of animals treated with a given packaging, Nb_Syr_Pack is the number of syringes in the packaging considered, and Nb_Syr_Trt is the number of syringes required for the whole treatment as indicated by the SPC.

Then, PUR was calculated in euros per animal using Equation (5) as follows:

PUR (€/animal) = (Objective − Rebate)/(Nb_Trt × Objective/Price)(5)

Similarly, for vaccines, the PUR was calculated for 1 year of protection using Equation (6) as follows:

PUR(€/animal) = (Objective − Rebate)/(Objective/(Price_Dose × Nb_Doses)(6)

where Nb_Doses is the number of doses for annual protection, and Price_Dose is the price per dose.

### 2.3. Descriptive Analysis

First, a descriptive analysis was performed. The contracts were compared by year and company to understand how they were built and how the rates and types of conditions were determined. Dispersion graphs of the PUR on the rebate rates of all medicines were drawn separately and for all possibilities of rebate rates when several rebate rates were possible for a given medicine. When appropriate, a comparison of a group of medicines with similar indications was conducted to determine the temporal pattern of the combinations among rebate, objectives and PUR.

### 2.4. Analytic Statistics

Before the analytic step was performed, a second set of restrictions was applied (Figure 2). First, the observations obtained with rebates for multiple medicines defined in the contract were not considered in this second step to limit the assumptions being made. Second, specific medicines were excluded to exclude outliers or medicines with very different characteristics within each category of medicine (AMs, APs, AIs and VACs). An AM medicine with a mean PUR of €15 per 100 kg BW was excluded since it was in up to twice the average PUR range (€1–10 per 100 kg BW) of the other AMs. The higher PUR of this medicine was consistent with the specificity of its indication (mastitis treatment by parenteral route). Moreover, most objectives were within the range of € [0; 25,000], and other objectives were excluded (195 of 2320 observations). Finally, the medicines expressed as doses before 2010 had very low PUR (€1 vs. €3.25 per dose), supporting the exclusion of these 5 observations.

Then, the data were analysed with R software [27]. A linear regression was performed using the nlme package of R. The outcome variable was PUR, and the explanatory variables were objective, year, yearly revenue from the veterinarian office of each firm, type of medicine (general administration, intramammary syringe, vaccine or per weight dose) and type of rebate (medicine, range, or global). The variable type of PUR was also created (per 100 kg BW or per dose). A step-by-step procedure was used to include the explanatory variables one-by-one, and then, final multivariate models were proposed based on the Akaike information criterion (AIC) values. Both the medicine name and firm were considered random variables.

## 3. Results

### 3.1. Medicine Typology

**Figure 3:**
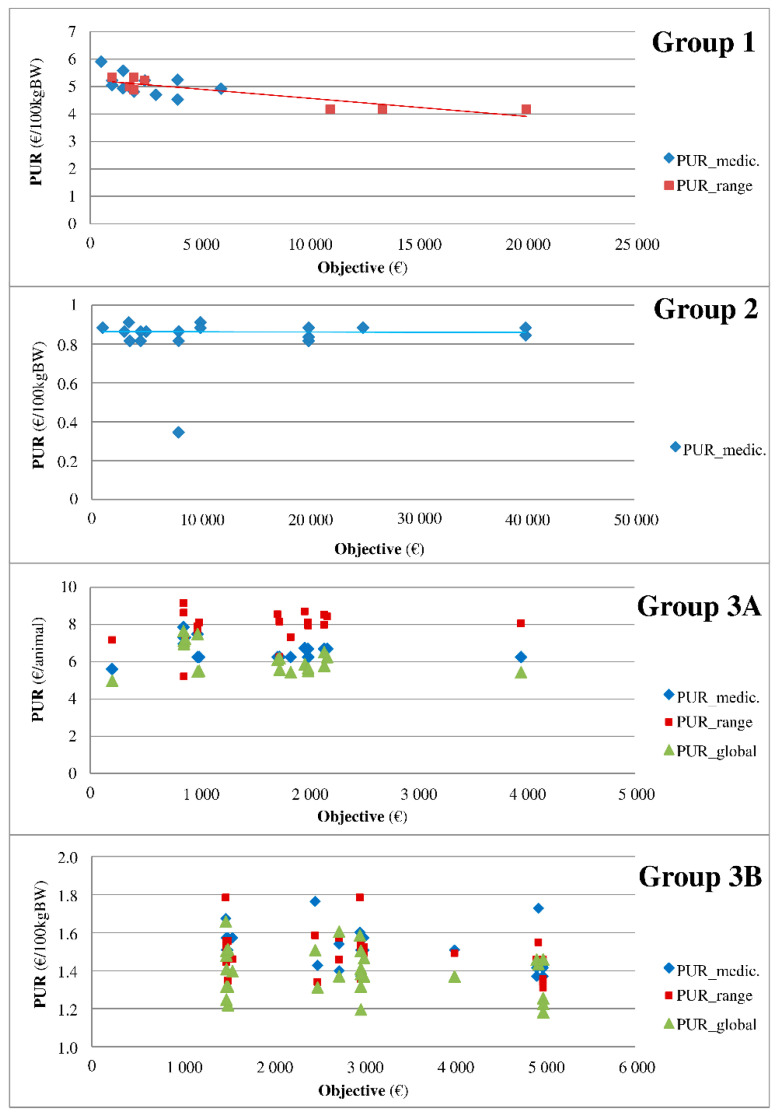
Typology of medicines according to the relationship between the PUR and purchase objectives. **Legend:** The medicines were classified into groups according to the relationship between the PUR and purchase objective as indicated in the contract. Group 1 includes medicines with a PUR that linearly decreases with the objective (the higher the purchase objective, the lower the PUR). The PUR does not change with the objective in group 2. Group 3 refers to medicines with 3 additive rebates and is divided into 2 classes. The PUR changes according to the type of rebate (medicine, range, or global) in group 3A, but such a relationship is not observed in group 3B.

The medicines were classified into five groups according to the relationship between the PUR and the purchase objective as indicated in the contract. Figure 3 summarizes the profile of each group, and the results of all medicines are shown in supplemental data 1 . Group 0 refers to medicines that are minimally represented in the sample (data not shown, n = 19). Group 1 includes medicines with a PUR that linearly decreases with the objective (the higher the purchase objective, the lower the PUR). The PUR does not change with the objective in group 2. Group 3 refers to medicines with three additive rebates and is divided into two classes. The PUR changes according to the type of rebate (medicine, range, or global) in group 3A, but such a relationship is not observed in group 3B. Finally, group 4 includes medicines with no relationship observed between the PUR and objectives.

### 3.2. Dynamics of Three Medicines with Similar Indications

**Figure 4:**
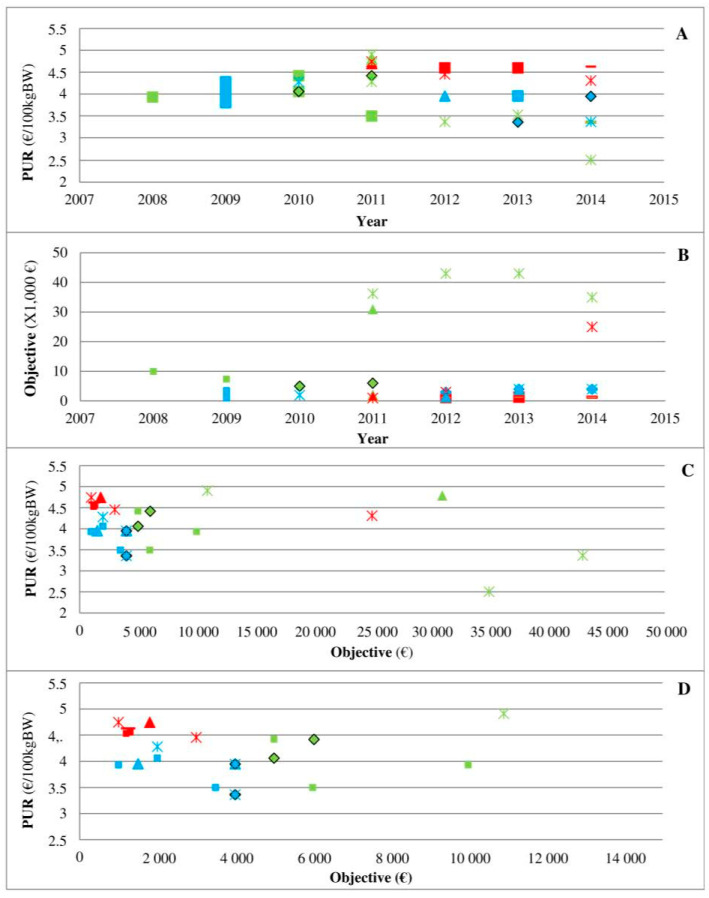
PUR depending on different years (**A**) and objectives ((**C**,**D**) for objectives <€15,000)) and PUR objectives depending on different years (**B**) for 3 medicines (C8P39 in green, C4P11 in blue, and C7P37 in red) and 5 offices (square, triangle, star, diamond, and dash). Legend: This figure presents 3 medicines indicated for respiratory diseases in cattle. The medicine C8P39 (green) arrived on the market in 2003, and its PUR was €4 to €5 per 100 kg BW up to 2010 (part A). The PUR of C4P11 (blue) was also approximately €4 per 100 kg BW up to 2010 (part A). The medicine C7P37 (red) arrived on the market in 2011, and a decrease in the PUR of C4P11 and C8P39 by €0.5 to €1 per 100 kg BW was observed among some veterinarians (part A). The objectives observed in contracts for C8P39 increased (Part B), whereas the objectives for the other 2 medicines remained very low. Part C described the relationship between the PUR and objectives with a focus on low objectives in part D.

Three medicines indicated for respiratory diseases in cattle (C8P39, C4P11, and C7P37 via their arrival on the market) were specifically analysed to better describe the place of the contracts in the veterinary-firm relationship (Figure 4). The medicine C8P39 (green) arrived on the market in 2003, and its PUR was €4 to €5 per 100 kg BW up to 2010 (Figure 4A). The PUR of C4P11 (blue) was also approximately €4 per 100 kg BW up to 2010 (Figure 4A). The medicine C7P37 (red) arrived on the market in 2011, and a decrease in the PUR of C4P11 and C8P39 by €0.5 to €1 per 100 kg BW was observed among some veterinarians (Figure 4A). This decrease in the PUR was achieved through an increase in rebates as follows: the data show that C8P39 used to have a rebate of 5–10% with objectives above €4000, whereas C7P37 and C4P11 arrived on the market with rebates of 10–25%. Then, the contracts observed for C8P39 reached 40%, but the objective was also increased (Figure 4B), whereas the objectives for the other two medicines remained very low. A rebate of 25% was finally offered to all veterinarians, i.e., with very low purchase conditions, by C7 for C7P37. Part C described the relationship between the PUR and objectives with a focus on low objectives in part D.

### 3.3. Factors Influencing the PUR: Analytic Statistics

**Figure 5:**
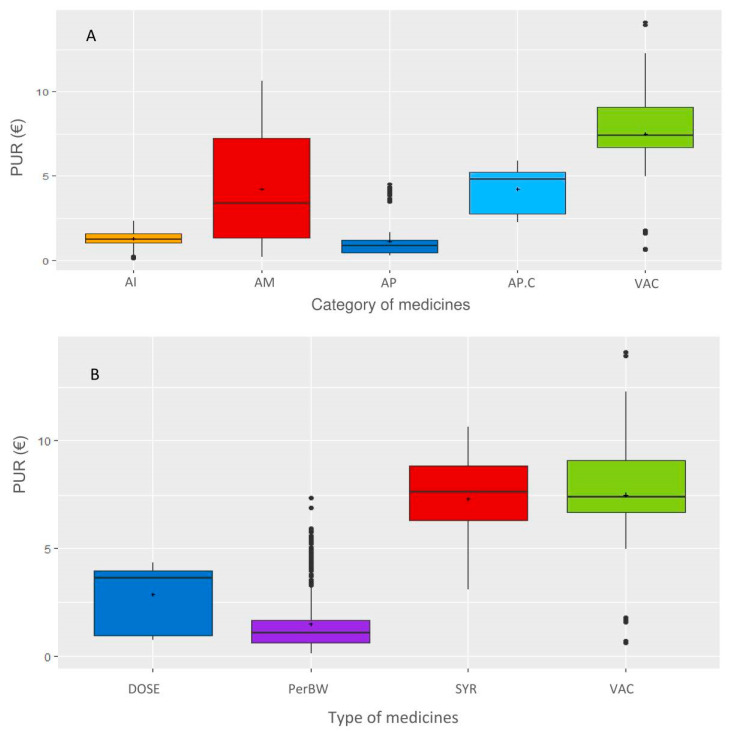
Distribution of the PUR in different categories (**A**) and types of medicines (**B**). Legend: The categories of medicines are as follows: AI: anti-inflammatories; AM: antimicrobials; AP: antiparasitics; AP.C: anticoccidials; VAC: vaccines; types of medicines are DOSE: per animal, Per body weight, SYR: syringes, and VAC: vaccines.

The distribution of the PUR per group and category of medicines is shown in Figure 5 and Table 1. AIs and APs have low variability, whereas AMs have large variability. Coccidiosis-related treatment was classified separately (AP.C) since its PUR is higher than that of the other APs. One medicine with a high PUR is an AP and is the only deworming medicine with a unique dose per animal (not per 100 kg BW). The medicine types VACs, SYR and, to a lesser extent, DOSE are higher than INJ, which is consistent with the PUR per animal for the first three types and per 100 kg BW for the fourth type.

The yearly revenue from the veterinarian office of each firm was not significantly associated with the PUR in any model. The average value of the PUR for a null objective, a medicine per 100 kg BW and the type of rebate for that medicine was €3.26 (Table 2). Compared to AMs, medicines from the categories AIs and APs were €2.1 and €2.0 lower than those from the category AP.Cs, respectively, and VACs were €1.1 and €2.3 higher, respectively. In the category AMs, an objective of €1000 was associated with a €0.023 decrease in the PUR, and a global rebate was associated with a €0.12 decrease in the PUR. Finally, AMs expressed per animal had a PUR that was €0.74 higher than that of AMs expressed per 100 kg BW. Moreover, the two by two and three by three interactions were significant, but the interpretations were complex. To better understand these interactions, an analysis was performed per category of medicines (Table 3, Table 4 and Table 5).

Regarding VAC, no explanatory variable was significantly associated with the PUR. The mean PUR was €5.28 per dose. Regarding AP.C, the average PUR was €3.50 per 100 kg BW for a null objective and a rebate on the medicine (Table 3). An extra objective of €1000 was associated with a €0.061 decrease in the PUR, and a global rebate was associated with a €0.97 increase in the PUR compared to a rebate on the medicine only. Regarding AI, the average PUR was €1.07 per 100 kg BW for a null objective and a rebate on the medicine (Table 3). An extra objective of €1000 was associated with a €0.029 decrease in the PUR, and a global rebate was associated with a €0.15 increase in the PUR compared to a rebate on the medicine only. No significant interaction was observed in AP.C or AI.

Regarding APs (Table 4), the average PUR was €1.15 per 100 kg BW for a null objective and a rebate on the medicine. An extra objective of €1000 was associated with a €0.0124 decrease in the PUR per 100 kg BW for medicines with a rebate on the medicine. The PUR of medicines with a PUR per animal was €1.48 higher than that of medicines with a PUR per 100 kg BW if all other variables remained constant. Thus, the PUR of medicines with a rebate on medicine for a null objective was €2.63 (i.e., 1.15 + 1.48) per animal. A global rebate was associated with a decrease in the PUR by €0.075 per 100 kg BW compared to a rebate on the medicine only, but the decrease in the PUR was €0.15 higher for medicines with a PUR expressed per animal and a global rebate compared to medicines with a PUR expressed per 100 kg BW and a medicine rebate. Moreover, the rate at which the PUR decreased was slower when the objective and a global rebate was applied (difference of €0.009 per €1000 of extra objective). As a result, for a medicine with a global discount, each €1000 extra objective was associated with a decrease in the PUR by €0.079 (−0.0124 − 0.0757 + 0.009) per 100 kg BW. Finally, each €1000 extra objective was associated with an average decrease in PUR by €0.072 per animal for medicine with a global rebate.

Regarding AMs (Table 5), the average PUR was €2.76 per 100 kg BW for a rebate on the medicine. Medicines with a PUR expressed per animal had a PUR that was €1.90 higher than the other medicines, leading to an average PUR of €4.66 per animal for a rebate on the medicine. A global rebate tended (*p* = 0.07) to be associated with an increase in the PUR by €0.20 per 100 kg BW compared to a rebate on the medicine only but was significantly associated with a €0.59 (−0.79 + 0.20) decrease in the PUR of medicines with PUR expressed by animal. Regarding medicines with the PUR expressed per 100 kg BW, the PUR was not associated with the objective but decreased by €0.031 per animal for each extra €1000 objective for medicines with a PUR expressed per animal.

## 4. Discussion

The present work represents the first study focusing on contracts between veterinary practitioners and pharmaceutical firms in the context of joint prescription and delivery. This study improves the understanding of the nature of the relationship between pharmaceutical firms and practitioner and quantifies the association between lower PURs and higher purchase objectives for different medicines.

### 4.1. Empirical Considerations

In all categories except for VAC, the objective is negatively associated with the PUR. Because the variables medicine and pharmaceutical firm were retained as random effects, this association indicates that, as expected, for a given medicine of a given firm, the real price paid by the veterinarian decreases when the objective increases. The decrease in the PUR for each extra €1000 of the objective ranges from €0.003 to €0.085 and even from €0.03 to €0.06 in most of the results. The present association is reported as linear since the other functions tested (squared, cube, etc.) were not significant. The relationship is unlikely to be linear as follows: a maximum rebate rate is observed for many medicines when the objective exceeds a threshold. Further research is needed to more precisely define the nature of the function linking the PUR and the objective. Even if the present study did not include real purchases but rather the objective of such purchases, the framework described here clearly demonstrates the relationship between the medicine price and quantity purchased by French veterinary practitioners during the studied period. The rate of contract completion is reportedly above 80% during this period. In summary, the decrease in the PUR for each extra €1000 of the purchase objective per category of medicine is established to be €0.061 per 100 kg BW for AP.Cs, €0.029 per 100 kg BW for AI, €0.0125 per 100 kg BW and €0.0845 per animal (only 1 medicine) for APs and €0.031 per animal for intramammary syringe AMs.

Interestingly, the PUR was not associated with the purchase objective of the vaccine, which is inconsistent with the expected results because vaccines represent a hot spot in the veterinary medicine market and yield a high revenue. Vaccines are often reported by field actors to be the subject of fierce competition in practice. The present lack of a significant association may be related to the fact that most observations (70%, i.e., 84 of 120 observations) were of the same pharmaceutical firm performing 3 additive rebates.

As expected, the PUR is negatively associated with a global rebate for APs and AMs, implying that the extra rebate reduces the PUR. However, this effect is limited to APs with a lower (even if negative) association between the PUR and purchase objective when a global rebate is given by the firm. The association between the PUR and purchase objective in the case of a global rebate is even lower (−€0.072) for animals as units of the PUR (compared to per 100 kg BW) probably because of the higher (+€1.48) average PUR for animals as units of PUR (compared to per 100 kg BW)

### 4.2. Principal-Agent Approach

The present results also provide new and clear insight into the respective positions of pharmaceutical firms and veterinarians in the French context. Agency theory considers the relationships between contractual parties as unequal as follows: the principal seeks to align the behaviour of the agent, who provides particular information, with his/her interests. In the present situation, considering the gap in terms of firm size, pharmaceutical firms have market power and, thus, are likely to be the principal, while veterinarians are likely to be the agent. Such a potential situation raised a key public health issue related to the prescription freedom of veterinarians in the context of joint prescription and delivery. Some of the present results support the consideration of pharmaceutical firms as the principal.

First, the marketing power of firms supports the idea that pharmaceutical firms are the principal. The results highlight the marketing efforts and imagination provided by pharmaceutical firms to present veterinarians with various types of contracts and different relationships between the rebate and objective, which may be considered a way to maintain information asymmetry. This variability includes different types of rebates, different periods of eligibility, and different ways to present the rebate obtained (percentage, absolute value, or free units). The different effect sizes of the regressions (€0.003 to €0.085 per extra €1000 of the objective) cannot be directly observed from the contract by the buyer, showing the strategy of pharmaceutical firms. The rebates defined in the contracts also follow multiconditional rules (multi-objective contracts), with varying conditions among different categories of medicines (three types of rebates) and even new extra conditions proposed during the year. This approach strengthens the intention of veterinarians to buy medicines from the same firm to increase the rebate and avoid any sharing of their purchases across different pharmaceutical firms. This approach also prevents any comparison by range of medicine or medicines technically equivalent (i.e., medicines with the same indication that are sold by two firms) by the veterinarian, reinforcing the firm as principal. Finally, the fact that the relationship between the purchase objective and rebate is limited to a maximum rebate rate per medicine or range of medicines clearly supports the pharmaceutical firm acting as the principal, which can even be observed as the final marketing strategy as follows: stimulating the purchase through rebates but limiting the overall amount of the rebate by complex rules may limit the understanding and overview of veterinarians regarding this question of prices.

Second, the analysis of three medicines in direct competition (Figure 4) clearly shows the marketing power of pharmaceutical firms and their ability to change the rules over time given the competition situation in the field. In a situation of an oligopoly, the medicine C8P34 has a low rebate that seems to be imposed by the firm to most clients. The ease of use (long-acting) and technical innovation may be reasons for its high demand, and the situation can be qualified as an monopoly since other medicines for the same indication have more difficult terms of use (twice daily administration, etc.). The medicine C7P37 arrived on the market with a high rebate, but its PUR remained the highest of the three medicines. Veterinarian decisions based only on rebates may lead to poor decisions, but any systematic transformation of rebates into the PUR remains very difficult due to heterogeneity across the contracts offered by pharmaceutical firms, which is a practice that reinforces information asymmetry. Interestingly, the first medicine on the market maintained the lowest PUR of the three medicines on the market throughout the period, highlighting the complex relationship between the PUR and objectives in cases of products with direct competition. Unfortunately, the present study did not allow us to perform a similar analysis of other medicines in direct competition due to inconsistency in contract collection and data availability.

Taken together, our results show that pharmaceutical firms can be considered the principal based on the information asymmetry and marketing power they can develop compared to the limited size of most veterinary offices. The bargaining power appears to be clearly unbalanced.

In contrast, other arguments support the consideration of veterinarians as the principal counteracting pharmaceutical firms’ power. In addition to the veterinary information superiority relative to farmers’ willingness to pay and farmers’ need for medicines, evidence from the present work suggests that veterinarians can be defined as the principal. First, the present work clearly highlights that veterinarians are mainly the price takers in cases in which pharmaceutical firms have a monopolistic position, but veterinarians become the principal in the case of oligopoly. Figure 4, which already shows the asymmetry of information implemented by pharmaceutical firms, also provides evidence of the place of veterinarians as the principal. When two new medicines arrived on the market (Figure 4), the shift from a monopoly (no real competitor of C8P34 with real innovation) to an oligopoly strengthened veterinarians through their prescription power to ensure that pharmaceutical firms change their financial conditions. This finding is consistent with a recent study that highlighted the change in AMU in cases of market changes (new medicines) at the national and regional levels, but this finding was observed only in medicines with similar medical indications (similar technical characteristics) [28]. Second, considering veterinarians the principal is reinforced by the low incitation given by pharmaceutical firms. The decrease in the PUR (€0.003 to €0.085 for each extra €1000 of the objective) is amazingly low, even though the absolute amount for veterinary offices can be high because of high revenue. Similarly, the positive association between the PUR and presence of a global rebate for AP.C, AIs and partly AMs can be interpreted as extra rebate in the case of a higher initial medicine price (likely innovative and/or recent medicine) that results from the bargaining power of veterinarians.

In summary, the present results demonstrate that veterinarians can be considered the principal once a monopoly on a medicine ends and remain the agent in cases of a monopolistic situation of a pharmaceutical firm for a given medicine. These findings are consistent with the literature extensively highlighting the major role of the market structure [29,30].

### 4.3. Policy Considerations

Agency theory highlights that both parties may have an interest in the principal compensating the agent in exchange for the abandonment of the informational advantage or consequences by the latter. Here, the situations may appear more complex as medicine is a regulated private good, and veterinarians jointly support public services through (i) the collective dimension of animal health, including zoonosis, (ii) limitation of the side effects of antimicrobial use, (iii) consolidation of animal and human welfare, and (iii) securitization of high-level service access in areas where it is limited.

There are increasing calls to separate delivery from prescription in veterinary medicine [7,8]. In the French context, such separation may challenge some public services currently provided by veterinarians because the loss of revenues generated from drugs sales could destabilize economic models of veterinary businesses. Such consequences need to be further studied, and further research is required to precisely define the joint support public services provided by veterinarians in different contexts (positive externalities). The present work shows that veterinarians remain the principal in the veterinarian-pharmaceutical firm relationship when no oligopoly is present for a given medicine (which is the case for many medicines). Consequently, there is no evidence from the present study that joint prescription and delivery may introduce any potential prescription bias linked to conflicts of interest under the market conditions during the 2008–2014 period.

The veterinary medicine market and institutional context of veterinary activities are changing very rapidly in France and Europe. First, the recent emergence of associations between practices and corporate veterinary groups [31] that share medicine purchases tends to reduce the bargaining power of pharmaceutical firms and can be considered a way to reinforce the position of veterinarians as the principal. Second, public incentives supporting veterinary presence in low density areas may bias the economic context in which veterinarians may work. These evolutions are challenging the state of the prescription delivery system described here, and agency theory could be reapplied to evaluate potential externalities linked to these new institutional contexts. Recent results from the application of transaction costs theory to the dairy sector [32,33,34] emphasize the contribution of the state to the legitimatization and improvement of the efficiency of contracts.

## 5. Conclusions

The present work represents the first study focusing on contracts between practitioners and pharmaceutical firms in the context of joint prescription and delivery. Even if pharmaceutical firms may appear as the principal, the evidence provided here warrants the consideration of veterinarians as the principal in the French context. The bargaining power between the two parties clearly appears to depend on whether the pharmaceutical firm has a monopolistic situation over a given medicine. The present study does not provide evidence suggesting that joint prescription and delivery may introduce any potential prescription bias linked to conflicts of interest under the market conditions during the 2008–2014 period.

## Figures and Tables

**Table 1:** Descriptive statistics of the PUR.

		PUR (€ per Dose or per 100 kg BW)			
		Unit	N	μ	σ
Category of medicine	AM	Both	530	5.01	4.38
	AI	€ per 100 kg BW	198	1.28	0.49
	AP	Both	440	1.25	1.40
	AP.C	€ per 100 kg BW	43	4.22	1.26
	VAC	€ per animal	119	8.91	3.59
Type of medicine	PerBW	€ per 100 kg BW	947	1.84	1.96
	DOSE	€ per animal	33	2.85	1.44
	SYR	€ per animal	226	7.07	2.19
	VAC	€ per animal	119	8.91	3.59

AM: antimicrobials; AI: anti-inflammatories; AP: antiparasitics; AP.C: anticoccidials; VAC: vaccines; PerBW: medicine administered with a dose per body weight; DOSE: medicine administered with a fixed dose per animal; SYR: intramammary syringe.

**Table 2:** Final linear regression of all groups (without interaction).

		Estimate in €	(SE)	*p* Value
Intercept		3.261	(0.284)	<2 × 10^−16^
Objective (per €1000)		−0.0234	(0.00306)	4.4 × 10^−14^
Category of medicine	AM	Reference		
	AI	−2.094	(0.815)	0.0117
	AP	−1.977	(0.567)	0.0007
	AP.C	1.167	(1.24)	0.3494
	VAC	2.380	(1.04)	0.0247
Type of rebate	Medicine	Reference		
	Range	−0.0702	(0.0882)	0.4265
	Global	−0.123	(0.0384)	0.0013
Unit of PUR	Per 100 kg BW	Reference		
	Per animal	0.7412	(0.269)	0.0060

The outcome variable is the PUR (€). SE: standard error; AM: antimicrobials; AI: anti-inflammatories; AP: antiparasitics; AP.C: anticoccidials; VAC: vaccines; the type of rebate can be applied to medicines only, a range of medicines or all medicines of a given pharmaceutical firm (global); per 1/100 kg BW: PUR expressed in € per 100 kg body weight; per animal: PUR expressed in € per animal.

**Table 3:** Final linear regression of anticoccidials (AP.C) and anti-inflammatories (AI).

		AP.C			AI		
		Estimate in €	(SE)	*p* Value	Estimate in €	(SE)	*p* Value
Intercept		3.50	(0.734)	0.0157	1.07	(0.168)	4.43 × 10^−5^
Objective (per €1000)		−0.0615	(0.011)	3.75 × 10^−6^	−0.0291	(0.003)	4.43 × 10^−15^
Type of rebate	Medicine	Reference			Reference		
	Range	−0.248	(0.131)	0.0656	0.156	(0.086)	0.0709
	Global	0.973	(0.289)	0.0017	0.144	(0.055)	0.0106

The outcome variable is the PUR (€). SE: standard error. The type of rebate can be applied to medicines only, a range of medicines or all medicines of a given pharmaceutical firm (global).

**Table 4:** Final linear regression of the category antiparasitics (AP).

		Estimate in €	(SE)	*p* Value
Intercept		1.15	0.0193	3.31 × 10^−6^
Objective (per €1.000)		−0.0124	0.00223	5.08 × 10^−8^
Type of rebate	Medicine	Reference		
	Range	0.0064	0.0622	0.917911
	Global	−0.0757	0.0213	0.000437
Unit of PUR	Per 100 kg BW	Reference		
	Per animal	1.48	0.0667	0.036096
Unit of PUR (per animal) × Type of rebate (global)		−0.146	0.0690	0.034093
Unit of PUR (per animal) × Objective (per €1.000)		−0.0132	7.986 × 10^−6^	0.100520
Type of rebate (global) × Objective (per €1.000)		0.0095	2.505 × 10^−6^	0.000171
Unit of PUR (per animal) × Type of rebate (global) × Objective (per €1.000)		−0.0717	1.791 × 10^−5^	7.57 × 10^−5^

The outcome variable is the PUR (€). SE: standard error. The type of rebate can be applied to medicines only, a range of medicines or all medicines of a given pharmaceutical firm (global); per 100 kg BW: PUR expressed in € per 100 kg body weight; per animal: PUR expressed in € per animal.

**Table 5:** Final linear regression of the category antimicrobials (AMs).

		Estimate in €	(SE)	*p* Value
Intercept		2.76	0.457	2.97 × 10^−5^
Objective (per €1.000)		−0.00721	0.0117	0.5411
Type of rebate	Medicine	Reference		
	Range	0.133	0.251	0.5972
	Global	0.200	0.110	0.0701
Unit of PUR	Per 100 kg BW	Reference		
	Per animal	1.90	0.324	9.45 × 10^−9^
Unit of PUR (per animal) × Type of rebate (range)		0.139	0.443	0.7538
Unit of PUR (per animal) × Type of rebate (global)		−0.788	0.134	9.31 × 10^−9^
Unit of PUR (per animal) × Objective (per €1.000)		−0.0031	1.335 × 10^−5^	0.0230

The outcome variable is the PUR (€). SE: standard error. The type of rebate can be applied to medicines only, a range of medicines or all medicines of a given pharmaceutical firm (global); per 100 kg BW: PUR expressed in € per 100 kg body weight; per animal: PUR expressed in € per animal.

## Data Availability

Data used for the present study are not public.

## Supplementary Materials

The following are available online at https://www.mdpi.com/2079-6382/10/2/176/s1, Supplementary material: Supplementary Data.
