# Supplementary material for: Could Contracts between Pharmaceutical Firms and French Veterinarians Bias Prescription Behaviour: A Principal-Agency Theory Approach in the Context of Oligopolies
Source: Antibiotics (Basel). 2021 Feb 10;10(2):176. doi: 10.3390/antibiotics10020176 (PMC7916326; doi:10.3390/antibiotics10020176)

Supplemental Data: drugs of group 1

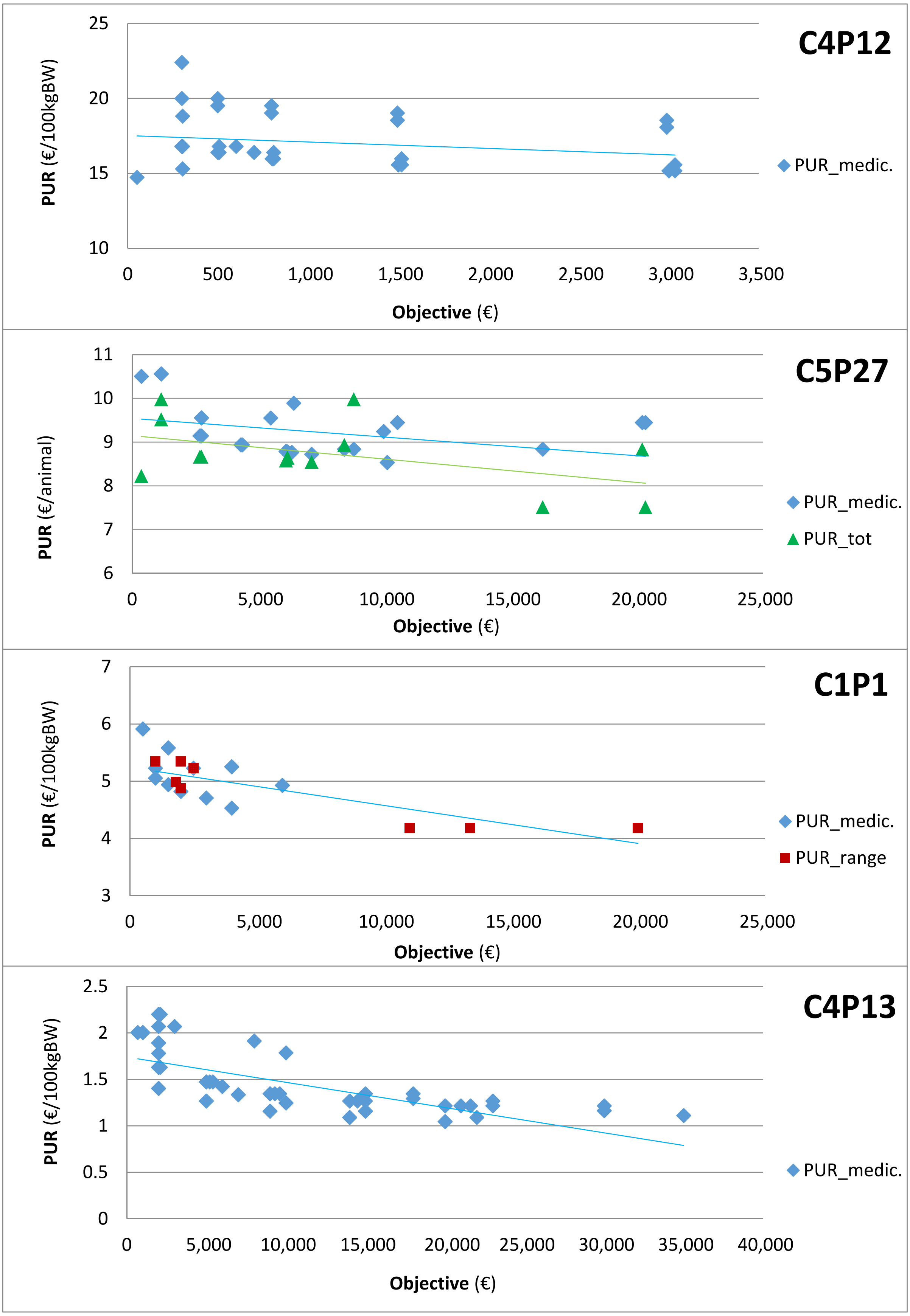

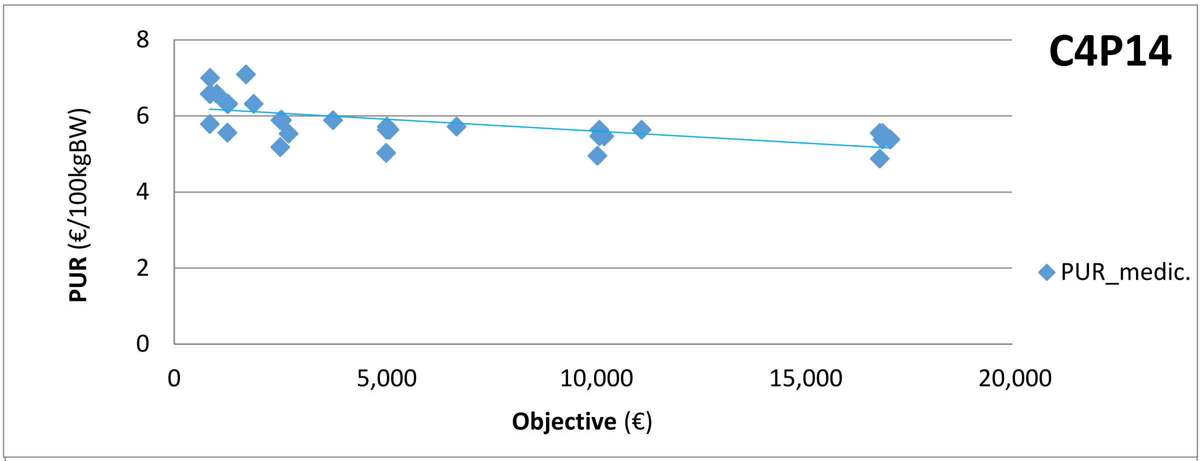
C4P14
PUR (€/100kgBW)
8
6
4
2
0
0
5,000
10,000
15,000
20,000
Objective (€)
PUR_medic.

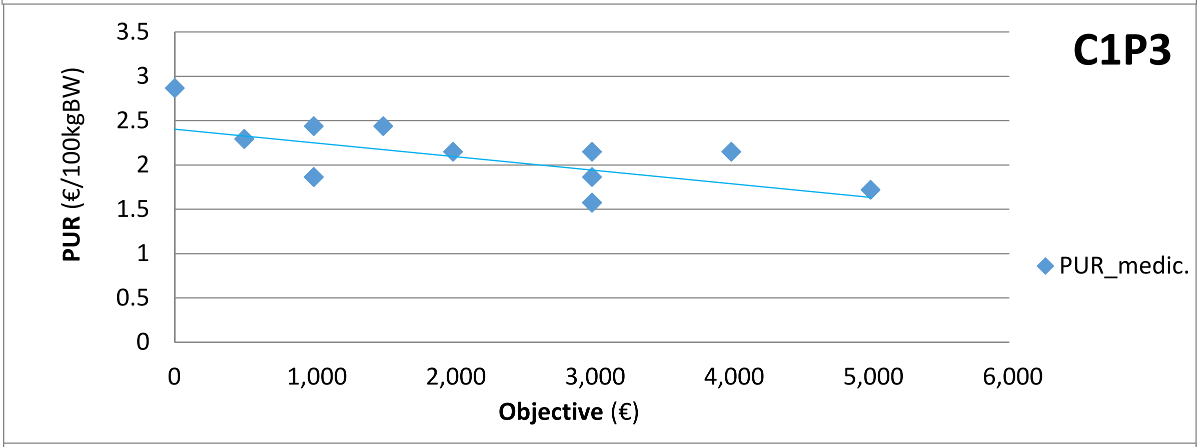
C1P3
PUR (€/100kgBW)
3.5
3
2.5
2
1.5
1
0.5
0
0
1,000
2,000
3,000
4,000
5,000
6,000
Objective (€)
PUR_medic.

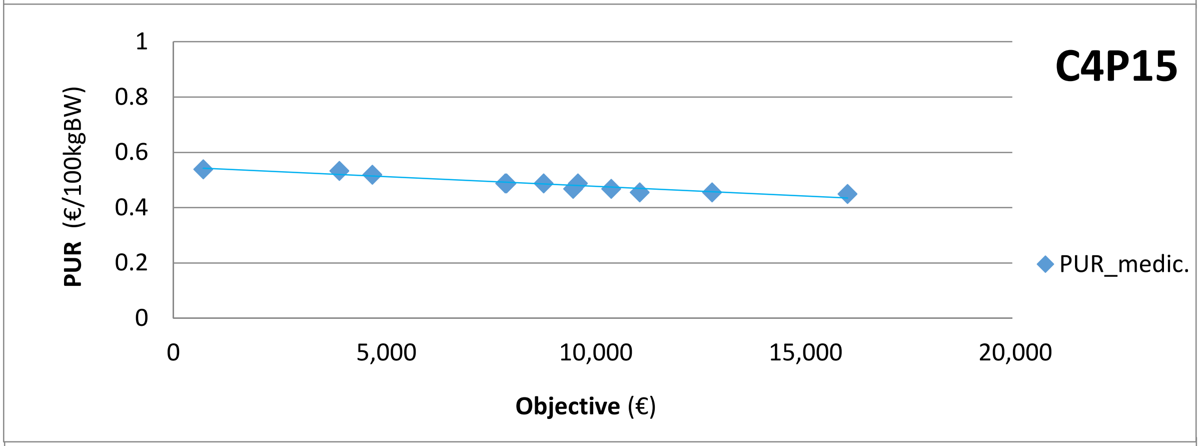
C4P15
PUR (€/100kgBW)
1
0.8
0.6
0.4
0.2
0
0
5,000
10,000
15,000
20,000
Objective (€)
PUR_medic.

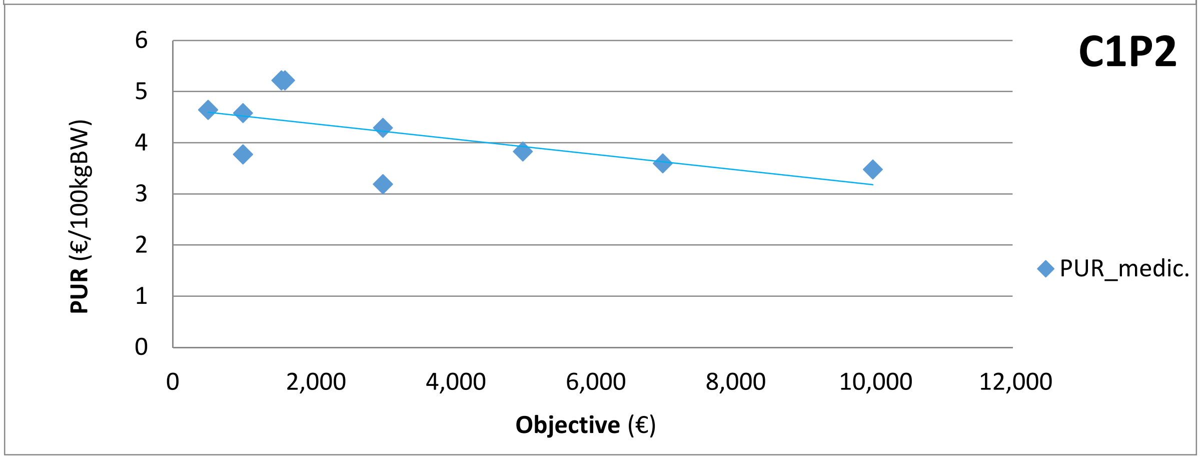
C1P2
PUR (€/100kgBW)
6
5
4
3
2
1
0
0
2,000
4,000
6,000
8,000
10,000
12,000
Objective (€)
PUR_medic.

Supplemental Data: drugs of group 2

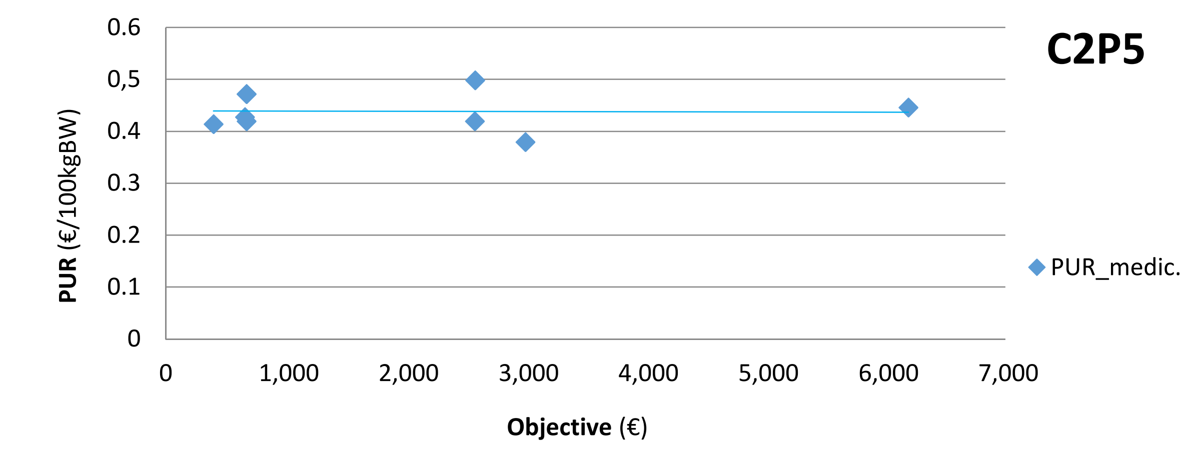

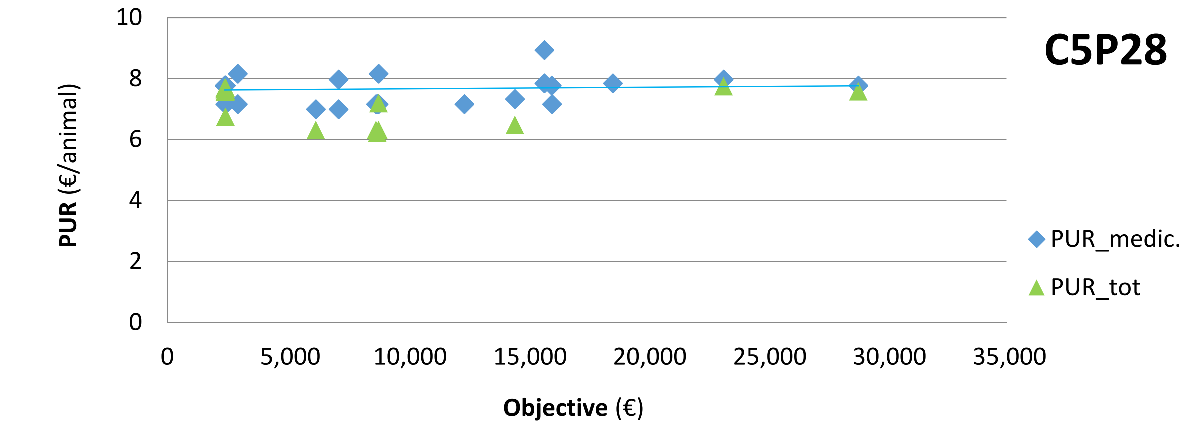

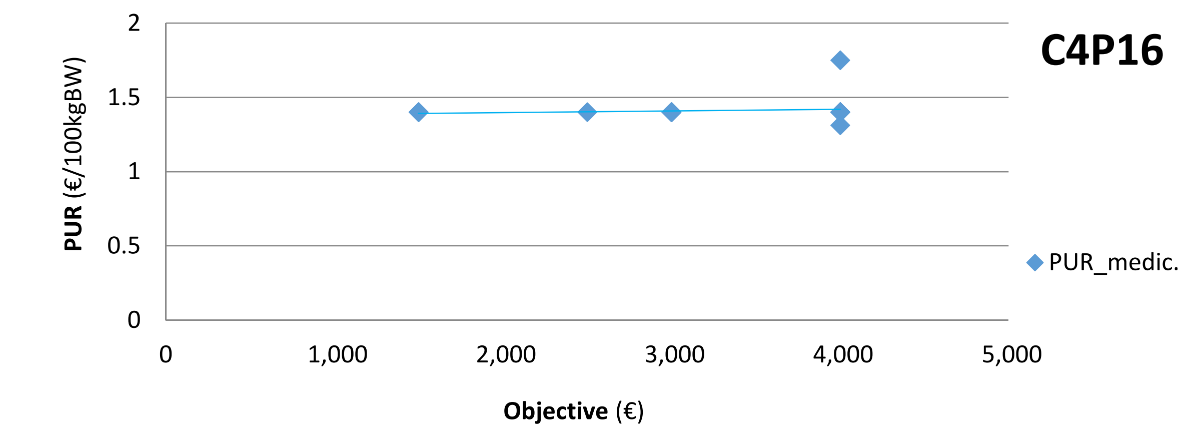

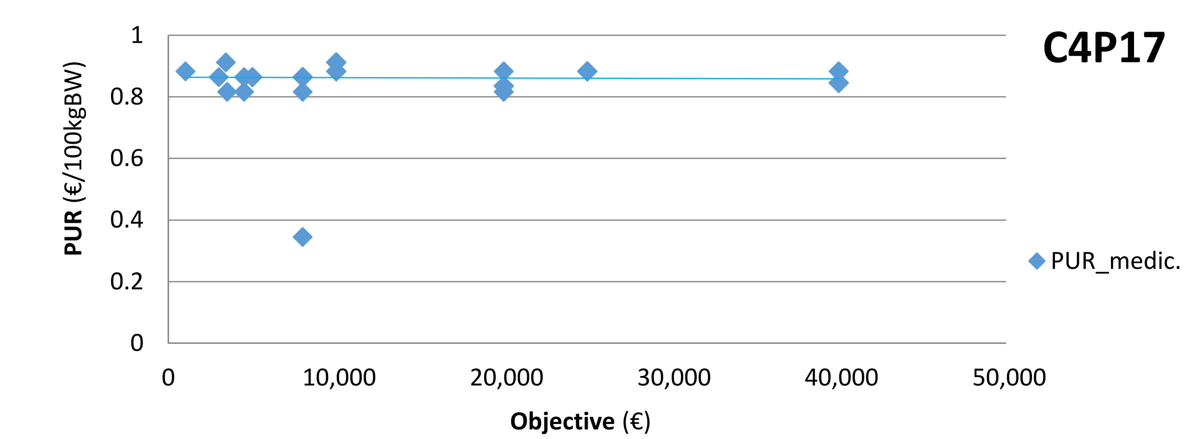

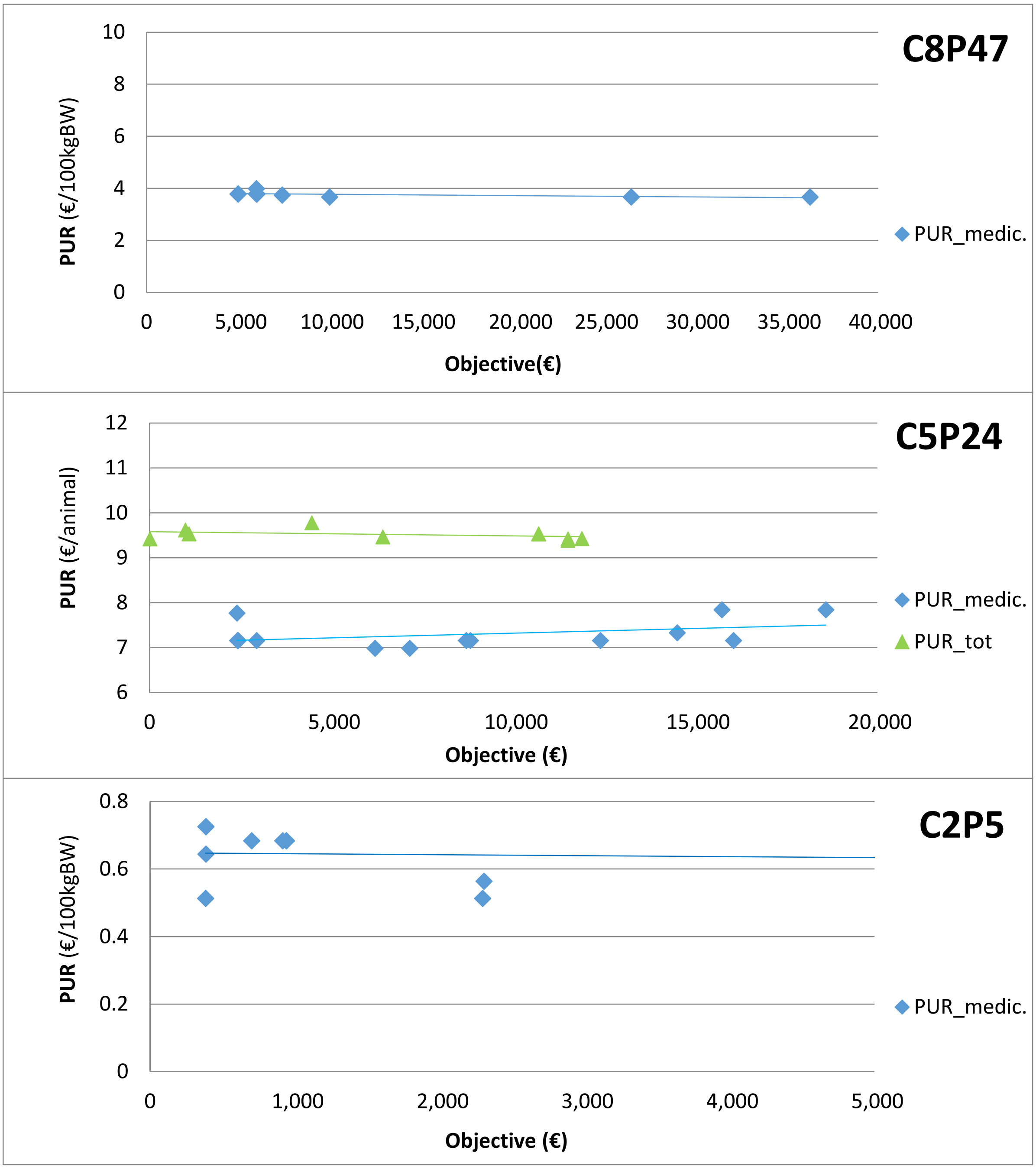
C8P47
PUR (€/100kgBW)
10
8
6
4
2
0
0
5,000
10,000
15,000
20,000
25,000
30,000
35,000
40,000
Objective(€)
PUR_medic.
C5P24
PUR (€/animal)
12
11
10
9
8
7
6
0
5,000
10,000
15,000
20,000
Objective (€)
PUR_medic.
PUR_tot
C2P5
PUR (€/100kgBW)
0.8
0.6
0.4
0.2
0
0
1,000
2,000
3,000
4,000
5,000
Objective (€)
PUR_medic.

Supplemental Data: drugs of group 3A

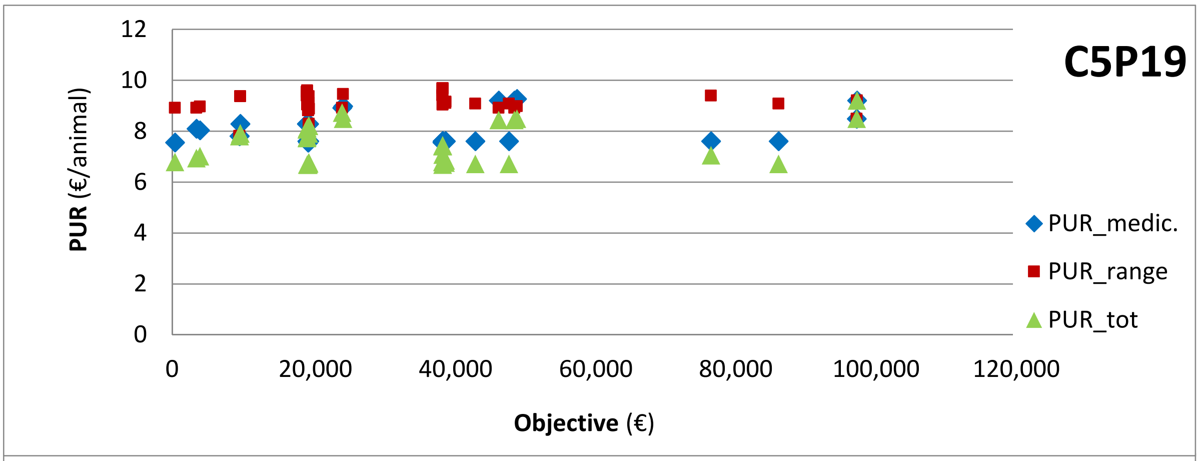

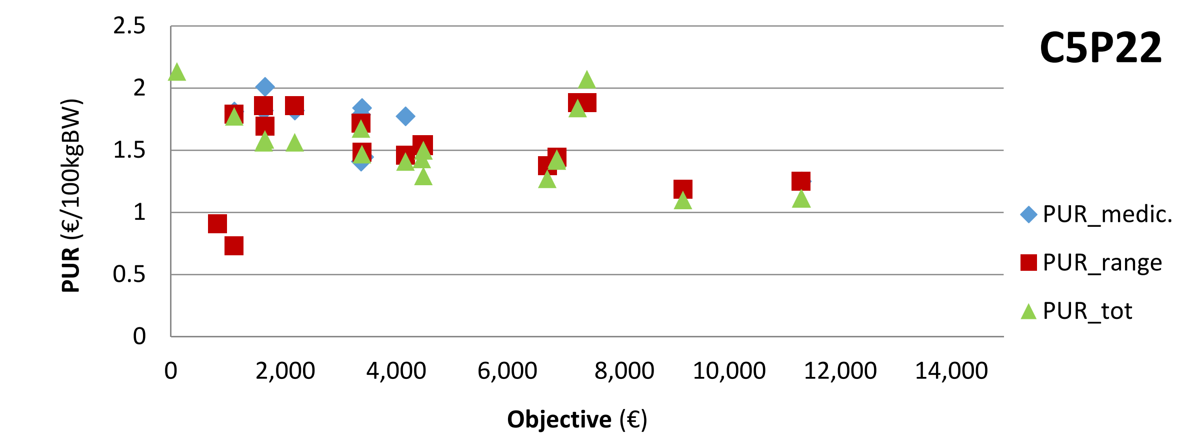

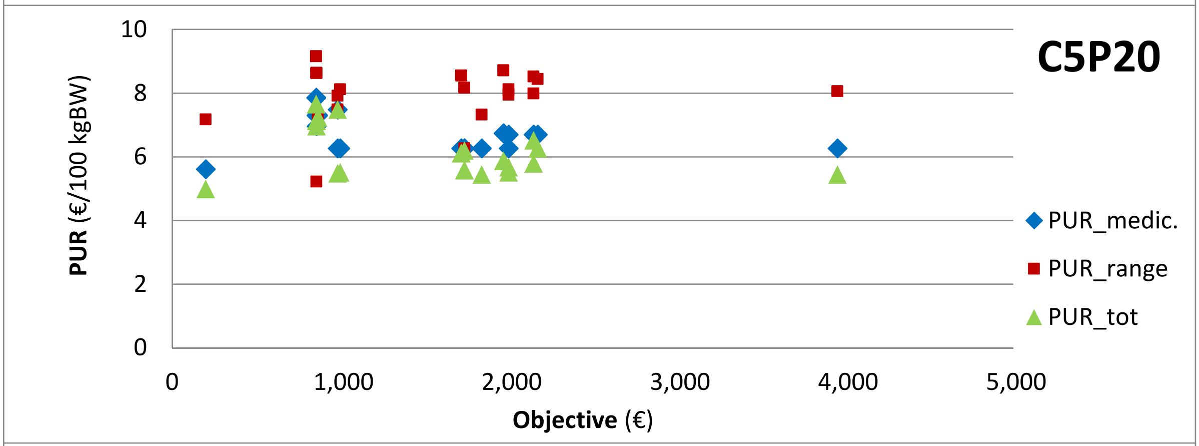

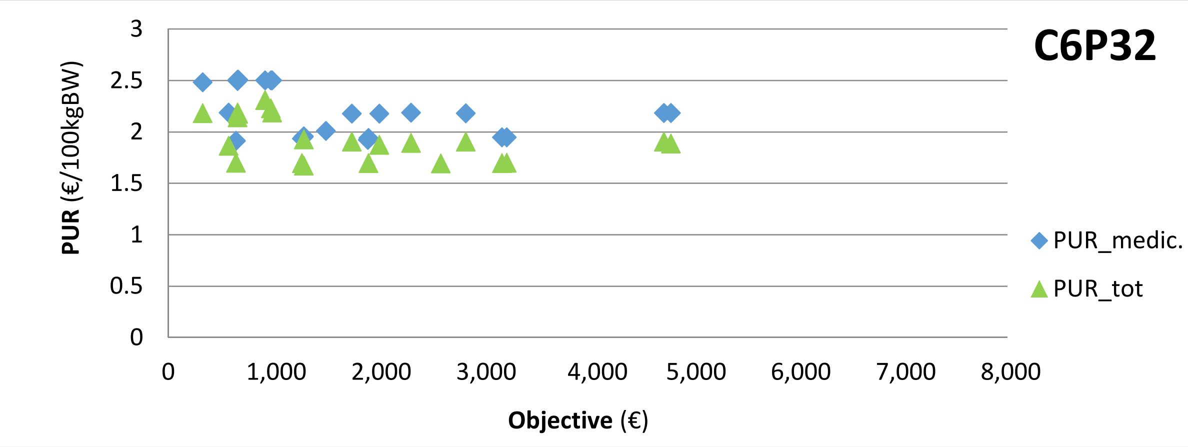

Supplemental Data: drugs of group 3B

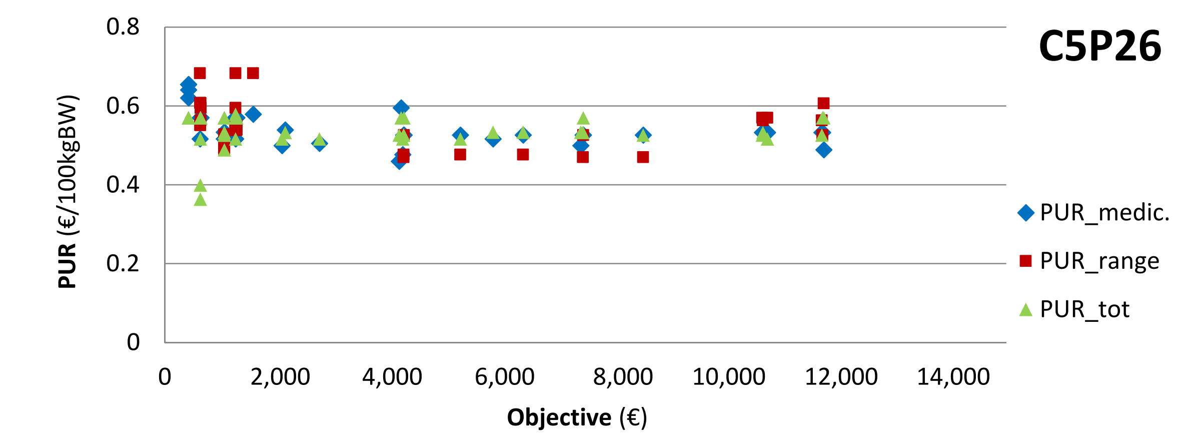

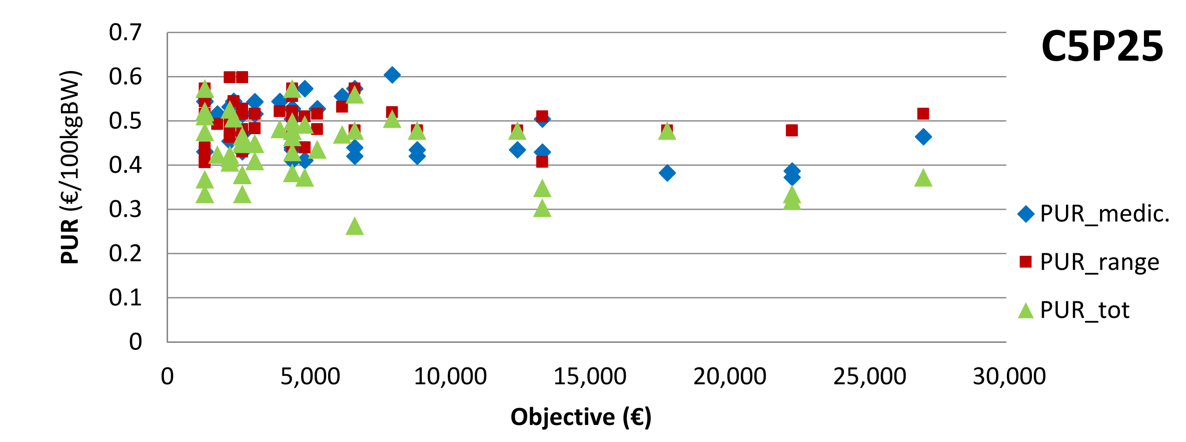

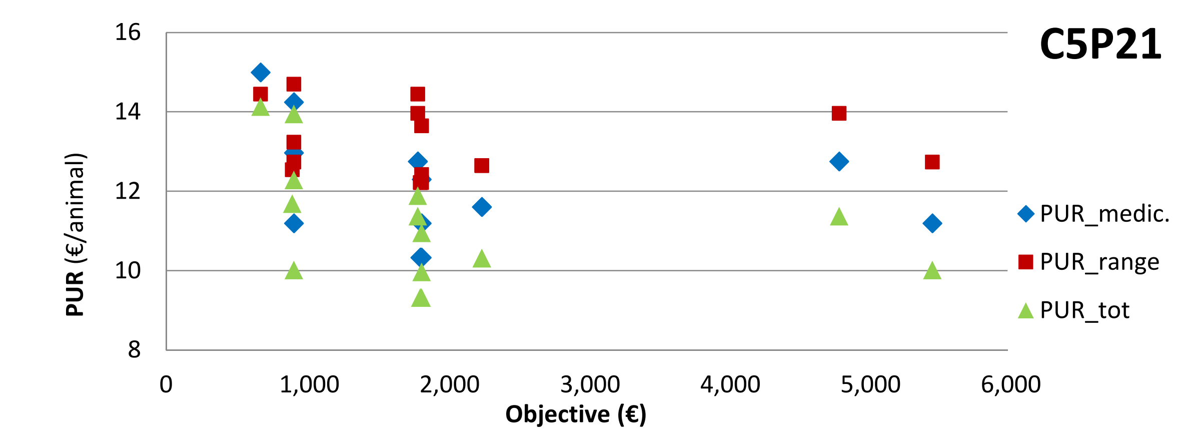

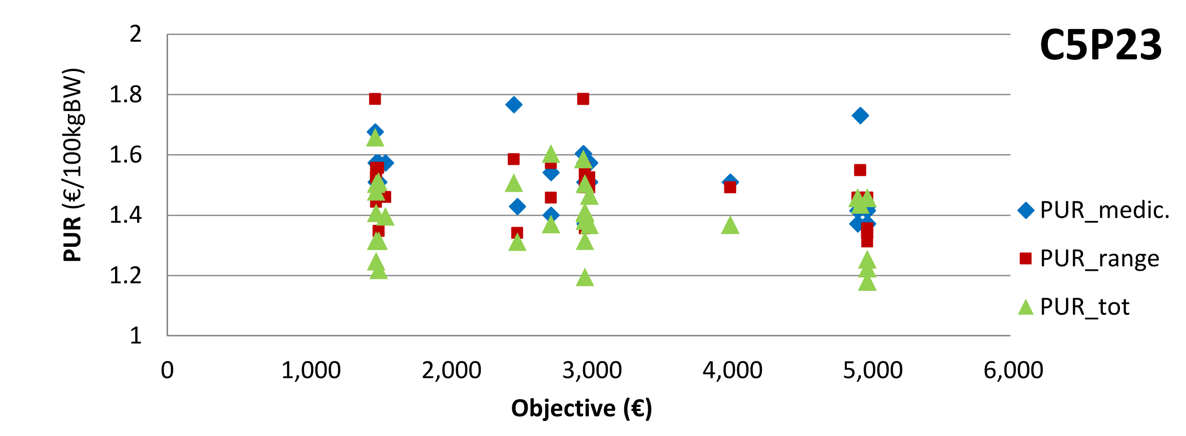

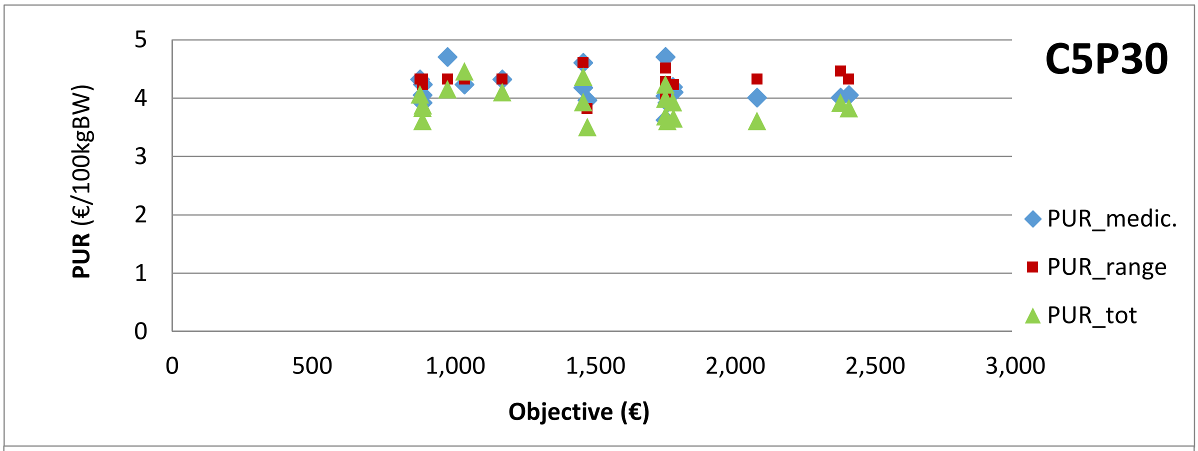
C5P30
PUR (€/100kgBW)
Objective (€)
0
1
2
3
4
5
0
500
1,000
1,500
2,000
2,500
3,000
PUR_medic.
PUR_range
PUR_tot

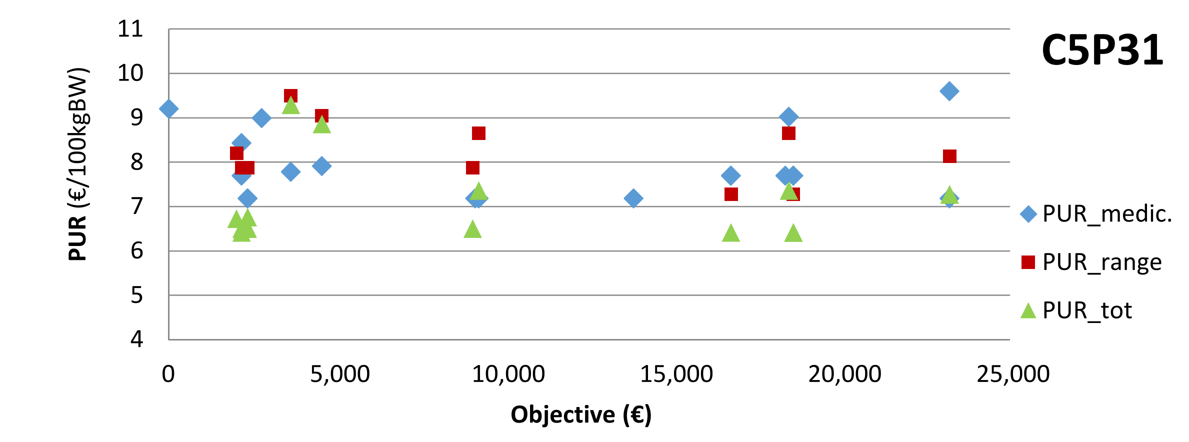
C5P31
PUR (€/100kgBW)
Objective (€)
4
5
6
7
8
9
10
11
0
5,000
10,000
15,000
20,000
25,000
PUR_medic.
PUR_range
PUR_tot

Supplemental Data: drugs of group 4

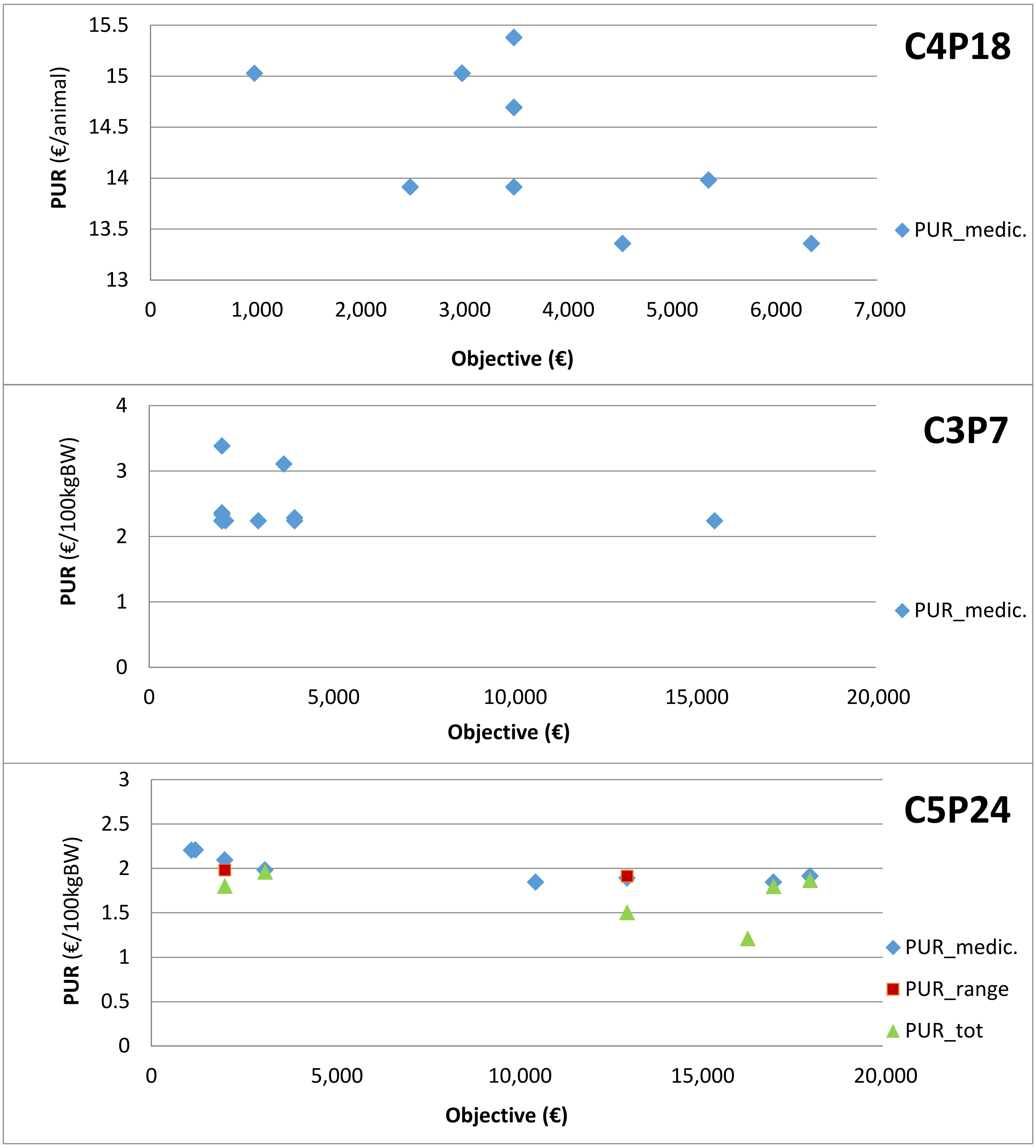

Supplement: Supplementary file 1 [file antibiotics-10-00176-s001.pdf]
